# The Potential Roles of Exosomes Carrying APP and Tau Cleavage Products in Alzheimer’s Disease

**DOI:** 10.3390/jcm12051883

**Published:** 2023-02-27

**Authors:** Yanfang Zhao, Yujin Gu, Qili Zhang, Hongliang Liu, Yingying Liu

**Affiliations:** 1 Institute of Biomedical Research, Shandong Provincial Research Center for Bioinformatic Engineering and Technique, Zibo Key Laboratory of New Drug Development of Neurodegenerative Diseases, School of Life Sciences and Medicine, Shandong University of Technology, Zibo 255000, China; 2 Institute of Translational Medicine, The Affiliated Hospital of Hangzhou Normal University, Hangzhou 310015, China

**Keywords:** Alzheimer’s disease, APP, Aβ, p-Tau, endosomal–lysosomal pathway

## Abstract

Alzheimer’s disease (AD) is the leading cause of dementia throughout the world. It is characterized by major amyloid plaques and neurofibrillary tangles (NFTs), which are composed of amyloid-β (Aβ) peptide and hyperphosphorylated Tau (p-Tau), respectively. Exosomes, which are secreted by cells, are single-membrane lipid bilayer vesicles found in bodily fluids and they have a diameter of 30–150 nm. Recently, they have been considered as critical carriers and biomarkers in AD, as they facilitate communication between cells and tissues by delivering proteins, lipids, and nucleic acids. This review demonstrates that exosomes are natural nanocontainers that carry APP as well as Tau cleavage products secreted by neuronal cells and that their formation is associated with the endosomal–lysosomal pathway. Moreover, these exosomes can transfer AD pathological molecules and participate in the pathophysiological process of AD; therefore, they have potential diagnostic and therapeutic value for AD and might also provide novel insights for screening and prevention of the disease.

## 1. Introduction

Alzheimer’s disease (AD) is the most common cause of dementia. It was first defined as ‘presenile dementia’ in 1901 by the German psychiatrist Alois Alzheimer. More than a century later, there are still no treatments that can cure or slow the progression of the disease. Current estimates suggest that 50 million people live with dementia worldwide at present. Globally, one new case of dementia is diagnosed every three seconds. The number of cases is expected to reach approximately 152 million by 2050 as the population ages. Approximately 50–60% of all diagnosed dementia cases are caused by AD. Moreover, the current annual cost of dementia is about USD 1 trillion per year, and this figure is forecasted to double by 2030, generating both a global health concern and a heavy economic burden [1].

AD is considered as a neurodegenerative disorder that is linked to aging, with the main characteristic being irreversible damage to neurons in the cortex, cerebellum, hippocampus, and basal cholinergic nuclei, which leads to the deterioration of memory as well as cognitive, mood, and behavioral functions [2,3]. However, no hypothesis has yet attempted to account for all symptoms and pathogenic mechanisms of AD. The senile plaques composed of amyloid β (Aβ) peptide and neurofibrillary tangles consisting of hyper-phosphorylated Tau (p-Tau) are commonly considered to be two key pathological features of AD [4].

## 2. The Aggregation and Accumulation of Aβ Peptides and Senile Plaques Is Linked to AD Pathology

The main component of senile plaques is the Aβ peptide, which is derived from the breakdown of amyloid precursor protein (APP). APP is a highly conserved, single-pass transmembrane protein with large extracellular domains, the expression of which is mainly localized on the cell membrane or in intracellular vesicles [5]. The APP gene is located on chromosome 21 and spans approximately 170 kb containing 18 exons. Alternative splicing of APP transcripts creates different mRNA isoforms ranging from 695 to 770 amino acids, of which the most 3 common isoforms are APP695, APP751, and APP770, all of which contain Aβ regions [6].

**Figure 1:**
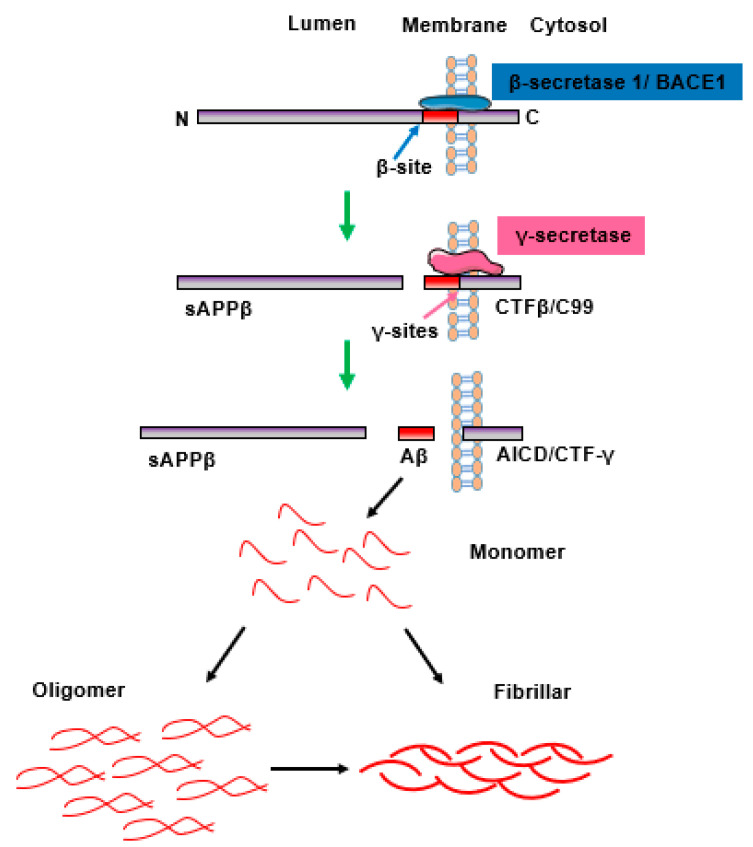
The process of the sequential cleavage of APP by the amyloidogenic pathway, which results in Aβ toxicity. APP is firstly cleaved by the protease β-secretase 1/BACE1 at the β-site, then releases sAPPβ (soluble APPβ) at its N-terminus and CTFβ/C99 at its C-terminus. The CTFβ fragment is sequentially cut by the γ-secretase/presenilin complex at the γ-sites, resulting in the formation of Aβ peptides. The Aβ peptides mainly containing Aβ_40_ and Aβ_42_ are more prone to aggregation and forming oligomers and fibrils, which causes toxicity to cells. BACE1: beta-site APP-cleaving enzymes.

Aβ is a 36–43 amino acids peptide that usually originates from the proteolytic processing of APP. Under physiological conditions, the level of Aβ in the brain maintains a balance in its production and clearance [7]. Under pathological conditions, APP is usually processed by the amyloidogenic pathway and releases neurotoxic Aβ fragments [7,8] (Figure 1). APP is first cleaved by the protease β-secretase 1 (beta-site APP-cleaving enzymes, BACE1, a membrane-spanning aspartyl protease with its active site located in lumen) at the β-site, thus releasing the majority of the extracellular portion of the protein at its N-terminus (soluble APPβ, sAPPβ) and a 99 amino acids long C-terminal fragment (C99, also called CTFβ). The CTFβ fragment is sequentially cut by the γ-secretase/presenilin complex at the γ-sites, resulting in formation of Aβ peptides, including those that have lengths of 40 (Aβ_1–40_ or Aβ_40_) and 42 (Aβ_1–42_ or Aβ_42_) amino acids [7,8].

The Aβ_40_ and Aβ_42_ fragment monomers generated from the cleavage of APP assemble to form a quantity of soluble oligomeric products [9]. These oligomeric species further self-organize and accumulate into sheet-like structures, called beta-sheets (β-sheets), then, each strand of β-sheet is assembled in parallel and polymerized with an alternate monomer. This ultimately leads to the formation of structurally distinct forms, including fibrils, protofibers, and polymorphic oligomers, referred to as β-plaques [10]. These become resistant to proteolytic cleavage and cause deterioration in neuronal health, leading to calcium homeostasis disequilibrium, oxidative stress, weakened energy metabolism and glucose regulation, cytokine release, inflammatory responses, and, eventually, neuronal cell death [8]. Moreover, Aβ and soluble Aβ oligomers can also affect normal learning and memory and impair cognition functions [11].

## 3. The Role of Hyperphosphorylated Tau and Neurofibrillary Tangles in the Progression of AD Pathology

**Figure 2:**
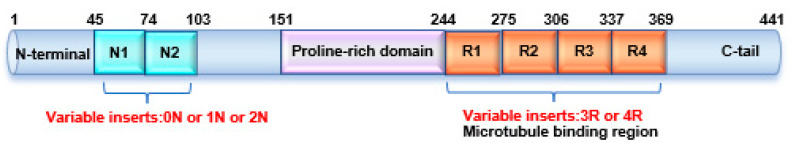
The domains of Tau proteins. Schematic representation of the longest Tau isoform: N1, N2 inserts in the N-terminal; proline-rich regions; the microtubule-binding domains: R1–R4 repeat inserts. N-terminal and microtubule binding domains are variable as the result of alternative splicing, and the variants contain 0N3R, 1N3R, 2N3R, 0N4R, 1N4R, and 2N4R Tau.

Intracellular neurofibrillary tangles (NFTs), which are mainly composed of hyperphosphorylated Tau, are considered one of the hallmarks of AD. Human Tau is encoded by the MAPT gene, which is located on chromosome 17q21.3 and composed of 16 exons [12,13]. In human CNS, the alternative splicing of exons 2, 3, and 10 yields 6 isoforms of Tau. These six isoforms vary from 352 to 441 amino acid residues and differ from each other according to the presence of 3 (3R) or 4 repeats (4R) situated at the C-terminal region and the 2 (58 amino acids, 2N), 1 (29 amino acids,1N) or no inserts (0N) in the N-terminal region of the Tau protein [14]. The N-terminal insert is known to modulate the interaction of Tau with neuronal membranes, and the repeat areas are responsible for binding microtubules [14]. The alternative splicing exclusion or inclusion of exon 10 generates Tau isoforms with 3R or 4R regions [14]. Normally, 3R-Tau and 4R-Tau are expressed in equivalent amounts in normal adult humans, with 3R-Tau isoforms mainly being generated in the developmental stage and 4R-Tau isoforms being generated in adulthood [13], whereas a changed ratio of 3R/4R-Tau occurs in most of the neurodegenerative Tauopathies [15] (Figure 2).

Studies have found that the main role of Tau in promoting microtubule assembly and stability is closely related to its post-translational modification phosphorylation. There are 85 potential phosphorylation sites located on the amino acid chain, and its phosphorylation state is linked to the biological activity of Tau proteins [12]. Under pathological conditions, the balance between phosphorylation and dephosphorylation of Tau is disrupted due to deregulation of the balance between kinases and phosphatases, which could result in the hyperphosphorylation of Tau [16]. The process whereby natively unfolded Tau becomes hyperphosphorylated is considered as a critical event in the progression of AD. The hyperphosphorylated Tau loses its affinity and dissociates from microtubules, becomes insoluble, and has increased expression in the cytoplasm; it is then prone to self-aggregation into PHFs, eventually forming NFTs [17]. The hyperphosphorylated Tau oligomers and NFTs exert various pathological effects, including axonal transport impairment, synapse loss, neuronal cytoskeletons, and mitochondrial dysfunction, as well as memory loss and cognitive lesions [17,18].

## 4. Exosomes

Extracellular vesicles (EVs) are natural nanoparticles that are secreted from unhealthy or dying cells in the body. EVs are categorized into three subtypes according to their diameter and biogenesis process, including exosomes (30–150 nm), microvesicles (MVs; 100–1000 nm), and apoptotic bodies (500 nm–2 μm) [19]. Exosomes are single-membrane lipid bilayer vesicles that have the same topology as a cell and are generated from the endosomal pathway either by vesicle budding into endosomes that mature into multivesicular bodies (MVBs) or by direct vesicle budding from the plasma membrane [20].

**Figure 3:**
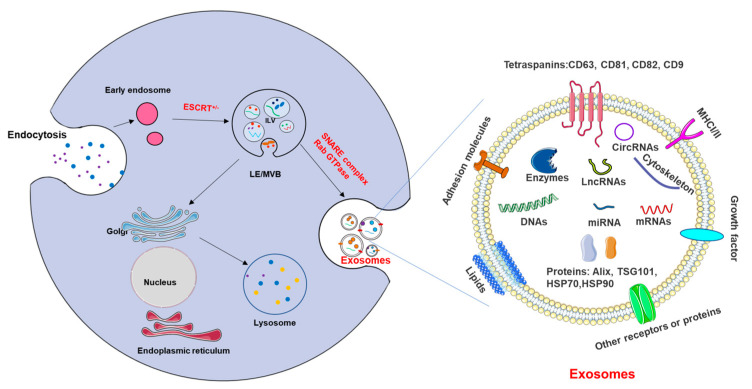
The process of exosome biogenesis and the cargos and markers of exosomes. Exosomes are generated from the invagination of the plasma membrane with cell−surface proteins and soluble proteins to form an EE and then converted to MVBs, including ILVs, through ESCRT−dependent (ESCRT^+^) or ESCRT−independent (ESCRT^−^) pathways. Rab proteins (Rab GTPase) participate in leading the MVBs toward the cell periphery; together with the SNARE complex, they facilitate MVB fusion with the plasma membrane, finally releasing ILVs, which now are called exosomes, into the extracellular space. The surfaces of exosomes contain many common molecules, including tetraspanins (CD63, CD81, CD82, CD9), MHCI/II, adhesion molecules, lipids, and cytoplasmic proteins such as enzymes, proteins (TSG101, Alix, etc.), miRNA, non-coding RNA, DNA, cytoskeletons, etc. ESCRT: endosomal-sorting complex necessary for transport.

Exosome biogenesis is controlled by precise biological modulation and is usually initiated by the activation of cell-specific receptors and their downstream signaling pathways [21]. Exosomes are generated when invagination of the plasma membrane with cell-surface proteins and soluble proteins forms an early endosome (EE), which then matures into a late endosome (LE). The inward budding of the LE membrane forms numerous intraluminal vesicles (ILVs) within MVBs (also termed multivesicular endosomes) through the presence of the endosomal-sorting complex necessary for transport (ESCRT) machinery [22]; alternatively, ILVs are sometimes formed through the absence of ESCRT (Figure 3). The main ESCRT apparatus major includes four complexes (ESCRT-0, -I, -II, and -III), the associated AAA ATPase vacuolar protein sorting 34 (Vps34) complex, and Alix. ESCRT-0 is responsible for recognizing the location of ubiquitylated proteins in the endosomal membrane and initiates the pathway. The combination of ESCRT-0, -I, and -II associated with ESCRT-III assemble to form a de-ubiquitination machinery, which packages cargo into maturing vesicles and advances vesicle budding into the luminal surface. This process is activated by phosphatidylinositol 3-phosphate (PIP3), hepatocyte-growth-factor-regulated tyrosine kinase substrate (HRS), the ubiquitination of the cytosolic tails of endocytic proteins, or curved membrane topology, and with the participation of Alix and TSG101 [23]. Subsequently, the transportation of MVB towards the membrane in the cytoskeleton and its fusion with the plasma membrane are mainly executed by Rab GTPases and the sensitive factor attachment protein receptor (SNARE) complex, ultimately releasing intraluminal vesicles such as exosomes [24]. Additionally, MVBs can be transported to the trans-Golgi network for endosomal circulation, finally combining with lysosomes or autophagosomes to be degraded [25]. The evidence suggests that MVB biogenesis can occur in an ESCRT-independent pathway, and the transportation of ILVs to MVBs can be triggered by lipid raft microdomains formed by ceramide and a tetraspanin such as CD63 [23].

After release into the extracellular environment, exosomes can target adjacent tissues and organs or be present in bodily fluids, including blood, cerebrospinal fluid, saliva, semen, urine, and breast milk, in order to transmit biological signals between parental or distant cells [26]. The cellular or organ targeting of exosomes is influenced by many materials, including receptors, transcription factors, enzymes, extracellular matrix proteins, lipids, and nucleic acids (mRNA and noncoding RNAs) inside and on the surface of the exosomes that constitute their content. Analysis of exosome contents reflects that some proteins specifically arise from parental cells and tissue and some are commonly found in exosomes [21]. The lipid bilayer membranes contain proteins such as tetraspanins (e.g., CD9, CD63, CD81, and CD82), which are a family of proteins characterized by the presence of four hydrophobic transmembrane domains, and conserved intracellular loops have been identified as relatively specific exosomal markers [27]. Moreover, TGS101, Alix, flotillin1, integrins, and cell adhesion molecules (CAM) are also considered as exosomal markers [28]. On the other hand, exosomes also contain a range of cytoskeletal proteins, such as actin, myosin, tubulin, fusion and transferring proteins, such as Rab2, Rab7, and annexin, and heat shock proteins, such as HSP70 and HSP90 [29]. Based on their size and protein and lipid content, exosomes can be analyzed using various techniques, including western blotting, flow cytometry, nanoparticle tracking analysis, mass spectrometry, and microscopy techniques [30]. In addition to lipids and proteins, various other genetic compositions, including mRNA, ribosomal RNA, microRNA (miRNA), long non-coding RNA (lncRNA), piwi-interacting RNA (piRNA), transfer RNA (tRNA), circular RNA (circRNA), and small Cajal body-specific RNA (scaRNA), have also been identified as existing in exosomes to execute cell-to-cell communication in different organs and tissues in the body [31]. The genetic materials and proteins in exosomes participate in normal physiological processes and diseases, and they are also regarded as biomarkers of various diseases [32].

The toxic Aβ and hyperphosphorylated Tau can be transmitted between cells and a substantial fraction of exosomes enter into second cells through an internalized dependent pathway [33], subsequently exerting toxic effects on the recipient cells and contributing to neuronal impairment in Alzheimer’s disease (AD).

## 5. The Propagation of Toxic Aβ Can Be Mediated by Exosomes in AD Pathology

### 5.1. Exosomes Acting as a Carrier to Transfer Neurotoxic Aβ between Neuronal Cells

EVs have been proven to be the location for the production and accumulation of APP-derived neurotoxic peptides. Meanwhile, EVs may also be useful for removing the neurotoxic peptides [34]. Exosomes have been presented as transporters for misfolded proteins; for instance, they feed more exogenous Aβ_42_, resulting in exosomes containing more Aβ_42_ from cultured cells [35]. Neuronal exosomes serve as transmitters to diffuse amyloidogenic peptides throughout the brain during the pathologic progression of AD. Exosomes extracted from peripheral plasma were injected into the mouse hippocampus and then observed to diffuse to other regions of the hippocampus and the cortex [36]. The increased level of Aβ oligomers in AD patients’ brains can be packaged into exosomes, which are subsequently internalized in cultured neurons and then their toxic content is transmitted to recipient cells. Furthermore, exosomes containing APP can spread to normal neurons in a dose-dependent manner [37], inhibiting the formation, secretion, or uptake of exosomes that could decrease the spread of toxic oligomers [38]. Neuroblastoma cells that express high levels of APP or APP Swedish mutation type (APPswe) were able to secrete exosomes containing APP and APP-derived products (CTFs or Aβ); these types of exosomes can be internalized, leading to the accumulation of pathogenic AD proteins in the receiving neuronal cells [39].

**Figure 4:**
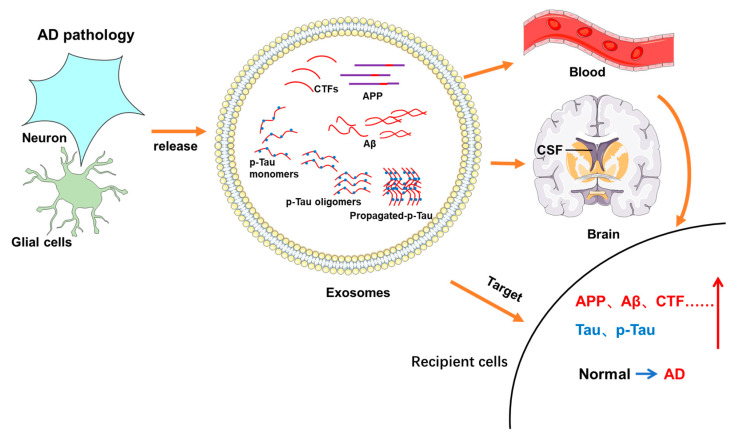
Exosomes acts as carriers, transferring neurotoxic APP and Tau derivatives between neuronal cells. The neurons or glial cells can release exosomes under AD pathological conditions, and the exosomes may contain APP, its cleavage products (CTFs or Aβ), and various forms of p-Tau, which can spread to recipient cells directly or indirectly by first entering the blood or CSF, ultimately leading to the accumulation of pathogenic AD proteins in receiving neuronal cells. CTFs: C-terminal fragments.

Several studies have considered the underlying mechanism whereby exosomes containing APP and APP-derived products spread to receiving cells (Figure 4) (Table 1). Treatment of exosomes derived from astrocytes with U18666, which can induce cholesterol sequestration, reduced exosomal release but enhanced levels of APP and its cleaved products in exosomes. Moreover, these exosomes could be internalized by cultured neurons in a phosphoinositide 3-kinase (PI3K)-dependent pathway and lead to neurotoxicity, contributing to the pathogenic progression of AD [40]. The deficiency of neutral sphingomyelinase 2 (nSMase2) can decrease the release of brain exosomes, the production of Aβ_42_, plaque deposition, and the overall brain amyloid load, resulting in enhanced cognition in 5XFAD mice [41,42]. The cellular prion protein (PrP^C^), which is highly abundant in exosomes and associated with oligomeric Aβ_42_, was found to drive Aβ fibrillization and prevent the neurotoxicity mediated by oligomeric Aβ_42_ in neuronal cells [43]. Moreover, Qin et al. found that PrPC could promote Aβ plaque deposition by enhancing the expression levels of APP [44]. These results prompted us to consider how PrP^C^ maintains a balance between neurotoxic and neuroprotective pathophysiology in AD and may also indicate a new approach for treating AD with respect to protein balancing. In summary, exosomes act as transporters for diffusing toxic Aβ and APP to recipient cells, and they are associated with the PI3K pathway, nSMase2, and PrP^C^.

### 5.2. The Interaction (Crosstalk) between Aβ and Glial-Cell-Derived Exosomes

In this context, the toxic influence of Aβ on neurons has been elaborated. However, the function of Aβ on glial cells also requires further exploration. Exposure to Aβ was able to decrease the secretion of exosomes derived from astrocytes, which was mainly caused by activation of the c-Jun N-terminal kinase (JNK) signaling pathway [47]. Aβ treatment can induce the selective release of small heat-shock protein HspB1 from astrocytes via a non-classical method of secretion. HspB1 was detected free in the medium or bound-to exosomes and was found to bind and sequester extracellular Aβ [48].

In addition, exosomes derived from astrocytes accelerate the development of AD under pathological conditions. The Aβ-related astrocyte-derived exosomes (ADEs) from the brain tissue and serum of a transgenic mouse model of familial AD (5 × FAD) or AD patients were rich in the sphingolipid ceramide, which promotes the binding of Aβ to voltage-dependent anion channel 1 (VDAC1) to form an oligomeric proapoptotic pore. This ultimately triggered downstream apoptosis, neuronal cell fragmentation, and death, which suggested that the neurotoxicity of Aβ was strengthened by exosomes [49]. Interestingly, ADEs under physiological conditions alleviate the progression of AD. ADEs contributed to a reduction in oligomeric Aβ-induced neurotoxicity in vitro and enhanced the clearance of Aβ in vivo. The production of exosomes secreted from astrocytes (ADEs) was strengthened by ultrasound, could be internalized by SH-5Y5Y cells, and reduced the uptake of Aβ_42_. ADEs can also be delivered into the brain and clear amyloid-β plaques across the BBB in APP/PS1 mice [50]. The astrocytes demonstrated a protective function on neurons by decreasing Aβ binding to oligomers and synaptopathy through the release of insulin and insulin-like growth factor-1 (IGF1) in an exosome trafficking pathway [51].

Moreover, microglia participate in the process of regulating the release of exosomes containing Aβ (Table 1). Microglia are major phagocytes in the brain and they participate in eliminating Aβ via the secretion of exosomes. The CHME3 microglia showed an early phagocytic influence on extracellular APP and Aβ aggregation and, later, release of inflammatory factors when co-cultured with SH-5Y5Y cells that overexpressed APP695swe. They could also internalize exosomes secreted from neuroblastoma cells and showed sustained sensitization to the overexpression of pro-inflammatory gene markers, implying that the dysregulation of the neuron-microglia signaling pathway participates in AD pathology [52]. The activation of microglia and the AD pathogenic process are linked to glutaminase C (GAC), which is upregulated in mouse brains with early AD. The enhanced expression levels of GAC promote the release of microglial exosomes and make functional alterations to exosomes and inclusions containing microglia-activated pro-inflammatory miRNAs in the early stage of AD pathogenesis [45]. The microglial transmembrane receptor TREM2 (a triggering receptor expressed on myeloid cells-2), which is located on the membrane of microglial exosomes, governs the release of exosomes, altering the inflammatory circumstances around Aβ and promoting the clearance of Aβ by microglia [53].

Thus, ADE promotes AD progression under pathological conditions while decreasing AD progression under physiological conditions. Moreover, microglia are involved in removing Aβ by releasing exosomes regulated by GAC and TREM2.

### 5.3. The Formation Process of Exosomes Containing APP and Its Cleavage Products Is Associated with the Endosomal—Lysosomal Pathway

A large body of evidence implies that the abnormal accumulation of APP-related products, such as CTF and Aβ, initiate intraneuronal communication in AD within vesicles of the endosomal–lysosomal (endolysosomal) pathway, which mediates both the generation and degradation of APP-derived products. Amyloid formation was proven to originate intracellularly, breaking the integrity of the intracellular membrane and resulting in lysosomal leakage [54]. The formation process of exosomes is associated with the endosomal–lysosomal pathway. These findings suggest that the metabolic process of APP and exosome production is involved in the process of sorting the endosomal–lysosomal pathway.

Several studies have explored the underlying molecular mechanism of endolysosomal system dysfunction for the formation of exosomes containing APP and its products. The post-translational modification ubiquitination of APP cytodomain lysines plays a critical function in APP endosomal sorting. Blocking the ubiquitination of APP by all of its mutant C-terminal lysines causes redistribution of APP from the endosomal intraluminal vesicles (ILVs) to the endosomal membranes; this is accompanied by a reduction in CTF levels but an enhancement of Aβ_40_ levels in secreted exosomes, mediated by presenilin 2 (PSEN2) cleavage [55]. The accumulation of CTFβ (C99) is closely associated with early lysosomal dysfunction [56]; treatment with γ-secretase inhibitor D6 caused further enhancement of endolysosomal-related APP-CTFs and aggravated the dysregulation of lysosomal-autophagic function [56]. Further studies revealed that D6 can facilitate APP-CTF oligomerization and induce oligomeric APP-CTFs to be mislocated in compartments of the endolysosomal network, including exosomes, by preventing C99 proteolysis that is dependent on the blockage of γ-secretase in APPswe-expressing cell media and the mouse brain. This suggests that D6 can be considered as a potential therapeutic strategy in AD pathology [57].

The components of the ESCRT apparatus are considered as critical regulators in the formation process of exosomes containing APP and its products. The secretion of CTFβ into small vesicles and the subcellular localization of APP are mediated by vesicle-related proteins Alix and Syntenin-1, the functions of which are important in the budding of the endosomal membrane. Meanwhile, the depletion of Alix and Syntenin-1 was able to change the subcellular localization of APP and attenuate the neurotoxicity induced by EVs containing APP [58]. The class III PI3K (PI3K-III)/Vps34 signaling pathway participates in the regulation of endolysosomal function and autophagy. Vps34 depletion can lead to endolysosomal membrane injury and advance the secretion of atypical exosomes that are rich in undigested lysosomal compounds, especially CTFs [59]. The accumulation and aggregation of Aβ have been observed in MVBs and can lead to the enlargement of MVBs in late endosomes, these events can be mimicked by the dysfunction of ESCRT-III as well as dominant negative VPS4A (dnVPS4A), suggesting that a deteriorating cycle of ESCRT-dependent late endosomal dysfunction is associated with Aβ accumulation [54]. Aβ accumulation can be induced by the interaction between APP and CD147, a subunit of γ-secretase, when its accessory protein Hook1 targets Rab22 in neuronal cells overexpressing APPswe695 under hypoxic conditions [60]. This evidence suggests that the endosomal–lysosomal system is dysregulated in AD pathology, and the substances that accumulate in neuronal cells are released into the extracellular space via EVs.

As well as Aβ being viewed as an endosomal–lysosomal route, the degradation of Aβ has also been linked with the exosome formation process. Intraneuronal Aβ can be degraded by metalloproteases, including endothelin-converting enzyme (ECE)-1 and -2. The activities of these enzyme were also detected in exosomes, implying that MVBs are intracellular sites of Aβ degradation. Moreover, prohibiting the activities of ECE proteins enhanced intracellular and extracellular Aβ aggregation and the intracellular generation of Aβ oligomers both in vitro and in vivo [61]. Moreover, Tetraspanin-6 (TSPAN6) is highly expressed in AD brains and acts as a crucial regulator in balancing lysosomal-dependent degradation and the secretion of exosomes enriched in APP-CTF. The overexpression of TSPAN6 can increase Aβ accumulation and shift the balance towards the generation of ILVs, eventually forming exosomes, while reducing the degradation of APP-CTF by impairing the autolysosomal pathway [62].

In brief, the main reason that the formation of APP-related products such as CTF and Aβ is toxic to the cell or causes Aβ degradation is closely linked to the formation process of exosomes in the endolysosomal pathway.

## 6. The Aggregation of Phosphorylated Tau Protein Can Be Regulated by Exosomes in AD Pathology

The propagation of pathological Tau proteins is a crucial characteristic of AD, and extracellular vesicles, especially exosomes, can spread this Tau pathology. For instance, exosomes can act as transporters for the spread of p-Tau pathology (Figure 4). Mice injected with plasma NDEs from ADC patients displayed increased p-Tau (PHF-1 antibody)-positive cells in the CA1 region of the hippocampus compared to plasma NDEs from patients with CNC and stable MCI patients [63]. Indeed, the exosomes extracted from the brains of Tau transgenic rTg4510 mice had high levels of Tau proteins, accelerated Tau phosphorylation, NFT production, and oligomeric aggregation, and could transfer the accumulation and misfolding of endogenous cellular Tau to recipient cells in a threshold-dependent way [64,65]. Tau in neuron-derived exosomes has different species, including monomers, oligomers, and aggregates, and the secretion of exosomes is raised by neuronal activity. These exosomes containing propagated Tau advance Tau accumulation and are associated with the trans-synaptic transmission of Tau between neurons [66]. Exosomes, taken from human induced pluripotent stem cell (iPSC)-derived neuron (iN)-conditioned media, cerebrospinal fluid (CSF), and plasma major, contained more mid-region-positive Tau than full-length (FL) Tau [67]. Although FL Tau has a greater tendency to propagate, the iN-derived exosomes containing Tau that were injected into wild mice possessed the ability to cause Tau aggregation and neurodegeneration, and they enhanced the dendritic blebbing of hippocampal neurons that were both ipsilateral and contralateral to the injection site in naïve mouse brains [68]. Moreover, mutant Tau expression in iNs caused the dysregulation of cargo proteins containing iN-derived exosomes, resulting in the aggregation of pathologic p-Tau after injection into the mouse brain [69].

Glial-derived exosomes enriched in Tau also contribute to Tau diffusion in the brain. The astrocytes were able to release Tau- and p-Tau-containing exosomes, which spread pathogenic Tau through the brain when exposed to Aβ_25–35_ but could be inhibited by the calcium-sensing receptor (CaSR) antagonist (calcilytic) NPS2143 [46]. The release of exosomes from the microglia transferred Tau and decreased Tau aggregation by restraining the synthesis of these exosomes both in vivo and in vitro [70]. The accumulation of Tau in exosomes was associated with microglia in Tau transgenic mice and could attenuate the accumulation of Tau by decreasing the release of exosomes [70].

In this context, we elucidated the interaction between Aβ aggregation and the endolysosomal pathway, which is also associated with Tau aggregation. The exosomal Tau escapes the endosome and propagation only occurs when the endosomal membranes present permeabilization, which is enhanced by the overexpression RAB7. This indicates the critical role played by the integrity of the endosomal membranes in transferring the aggregated protein to escape lysosomal degradation to recipient cells [71].

In summary, exosomes derived from neurons or astrocytes under AD pathological conditions can transfer and promote the accumulation of p-Tau, while exosomes from microglia can decrease aggregation. Moreover, the biogenesis of exosomes containing Tau is also modulated by the endosomal–lysosomal pathway.

## 7. Exosomes including Aβ and Tau Acting as Biomarkers in the AD Pathological Process

Currently, numerous clinical evaluations, including cognitive tests and functional brain imaging (MRI, PET, and SPECT scans), are employed to evaluate AD [72]. Moreover, detecting markers, including Aβ_42_ (Aβ_1–42_), total-Tau (tTau), and p-Tau-181, in CSF is paving the way for AD diagnosis based on biomarkers [73]. A growing body of evidence suggests that the crucial functions of exosomes that contain certain aggregation-prone proteins, including Aβ, APP C-terminal fragments, Tau, and the prion protein, are involved in the pathophysiology of AD [74]. The aggregation-prone proteins transfer from cell to cell and spread across the brain via transportation by vehicles, leading to onset and propagation of the disease [75]. Furthermore, the pathogenic proteins in exosomes derived from brains containing AD were proven to be able to freely cross the blood–brain barrier (BBB), thus suggesting that brain-derived exosomes could be potential biomarker carriers for AD in the blood [76].

**Figure 5:**
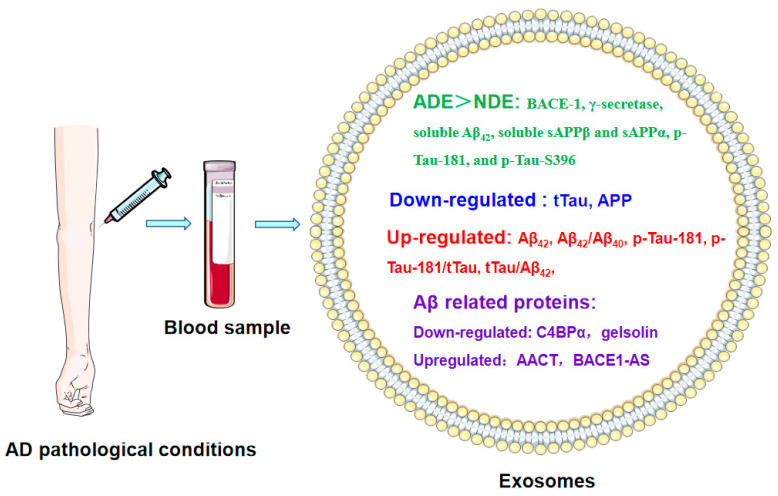
The derivatives of Aβ and Tau were identified as biomarkers in plasma exosomes under AD pathological conditions. The levels of tTau, APP, C4BPα, and gelsolin were downregulated while levels of Aβ_42_, Aβ_42_/Aβ_40_, p-Tau-181, p-Tau-181/tTau, tTau/Aβ_42_, AACT, and BACE1-AS were upregulated in plasma exosomes in cases of AD. In addition, the levels of ADE cargo proteins, including BACE-1, γ-secretase, soluble Aβ_42_, soluble sAPPβ and sAPPα, p-Tau-181, and p-Tau-S396, were significantly higher in ADE exosomes of AD patients. AACT: alpha-1-antichymotrypsin.

Determination of the pattern of several AD-related proteins in plasma exosomes has value for exploring blood-based biomarkers at different stages of AD. Plasma exosomes were demonstrated to be ring-shaped in both AD patients and healthy controls; however, compared to plasma exosomes in healthy people, the exosome distribution in individuals with AD was more concentrated and the exosome diameter was relatively smaller [77]. To be protected from degradation by the bilayer membrane in exosomes, AD pathogenic proteins were more enriched in plasma exosomes than in plasma [76]. Tau in central-nervous system (CNS)-derived exosomes, which are labeled by a putative CNS-specific marker-L1 cell adhesion molecule (L1CAM), have been found to be readily transported from the brain to the peripheral blood in AD mice [78]. The concentrations of tTau and APP were reduced while the p-Tau-181/tTau ratio, Aβ_42_ level, Aβ_42_/Aβ_40_ ratio, and tTau/Aβ_42_ ratio were increased in plasma EVs, including exosomes, in the mild and moderate stages of AD [79]. Furthermore, the raised levels of p-Tau-181 and the tTau/Aβ_42_ ratio in plasma EVs were both negatively correlated with cognitive scores [74,76]. Moreover, older participants with cognitive decline less severe than MCI or dementia also presented higher levels of total Tau, p-Tau-181, and p-Tau-231 in neuronal extracellular vesicles (nEVs) than cognitively stable participants [80]. Plasma neuron-derived exosomal levels of p-Tau-181 and Aβ_42_ changed markedly with increasing age in AD patients compared to controls, while there was no alternation in p-Tau-S396 levels [63,81]. The difference between Aβ_42_, tTau, p-Tau-181, and p-Tau-S396 levels in plasma neuron-derived exosomes in control, aMCI, and AD individuals was strongly related to CSF levels, and the diagnostic powers of these combined markers in exosomes were similar to those of CSF. Moreover, the AUC (area under the curve) values of these combined markers in exosomes and CSF were both higher than those of individual markers, suggesting that this combination of exosomal biomarkers had higher diagnostic efficiency than each single biomarker, and the exosomal biomarkers had the same diagnostic power as the CSF biomarkers [77,82]. The combination of Aβ_42_ in plasma NDEs and Sniffin’ stick (SS-16) scores exhibited better prediction of the conversion of MCI to AD dementia at two- and three-year return visits [83]. In summary, AD is a disease that progresses from an asymptomatic phase, to a minor cognitive (MCI) phase, to AD with mild, moderate, and severe dementia phases with biomarker evidence [84], including higher levels of exosomal Tau, p-Tau-181, and p-Tau-231. This is followed by the MCI phase, which is characterized by Aβ_42_ and enhanced levels of tTau and p-Tau-181; meanwhile, high levels or ratios of p-Tau-181/tTau, Aβ_42_, Aβ_42_/Aβ_40_, and tTau/Aβ_42_ are seen in the mild and moderate AD phases. Moreover, these complex pathogenic AD proteins found in plasma neuronal exosomes have been proven to be more dependable biomarkers for AD than single markers alone [74] (Figure 5).

In addition, other Aβ-peptide-related biomarkers involved in the progression of AD pathology were also identified in exosomes. For instance, levels of two Aβ-binding proteins, including alpha-1-antichymotrypsin (AACT), were increased and the level of C4b-binding protein alpha chain (C4BPα) was decreased in plasma exosomes in a case of AD; these proteins were therefore identified as potential exosomal biomarker candidates for AD diagnosis [85]. The levels of gelsolin, which is related to restraining Aβ fibril formation, were lower in serum exosomes in patients with dementia than in controls [86]. The level of BACE1-antisense transcript (BACE1-AS) was notably increased in plasma exosomes in AD patients compared with the normal controls [87]. However, Fotuhi et al. found no notable differences between levels of BACE1-AS in the plasma exosomes of AD and control participants. The evidence suggests that more samples need to be evaluated to verify the different data.

Interestingly, the composition of the Aβ_42_-generating system was detected in two separate, independent sets of astrocyte-derived exosomes (ADEs) and NDEs in the plasma of AD patients and controls. The levels of β-site amyloid precursor protein-cleaving enzyme-1(BACE-1), γ-secretase, soluble Aβ_42_, soluble sAPPβ and sAPPα, p-Tau-181, and p-Tau-S396 were dramatically higher (3- to 20-fold) in ADEs than in NDEs in both AD patients and matched normal subjects, which suggests that ADE cargo proteins may facilitate an investigation of the mechanisms of cellular interactions and biomarkers in AD [88]. Moreover, Tau levels were found to be enhanced in the microglia-derived EVs of AD patients compared with controls [89]. Animal experimental data also confirmed that the levels of Aβ and Tau in plasma NEVs and AEVs were consistent with their levels in the brain [90]. These data demonstrate the potential role of microglia-derived EVs in the spread of Tau in the human brain and the progression of AD pathology.

As well as exosomes that contain pathogenic proteins as markers extracted from CSF and plasma, several studies have demonstrated the novel detection of vehicle-based urinary or salivary exosomes in the early stages of AD. The levels of AD pathological proteins in urinary exosomes of AD patients were significantly higher than those found in matched healthy controls. The quantity of urinary exosomes in AD patients was also higher than that in healthy subjects assessed by NTA [91]. The expression levels of Aβ oligomer/fibril, Aβ, and phosphorylated Tau contained in salivary exosomes were markedly higher in AD and cognitively impaired patients compared with healthy subjects, a finding that contributes to research into the pathological progression of AD [92].

## 8. Conclusions

Exosomes originate from various cells under specific physiological or pathological conditions. However, to date, the exact functions of exosomes have still not been sufficiently explained. This review demonstrated that exosomes act as major natural nanocontainers containing APP cleavage products as well as hyperphosphorylated Tau, which is secreted by neurons and microglial cells. The formation process of these exosomes are involved in the endosomal–lysosomal pathway; furthermore, they can transfer AD pathological molecules and are closely associated with the pathological processes of advanced AD.

Exosomes originate from the body’s cells, meaning that their contents are tightly associated with the functional state of the donor cell. Moreover, exosomes are characterized by their smaller size, more extensive sources, and lower immunogenicity compared with donor cells. This suggests that exosomes can be considered a potential and promising noninvasive biomarker in the diagnosis of a variety of diseases, including AD. The cause of most AD cases remains unknown, except for genetic mutation, but several important mechanisms have been explored as potential causes of AD, including Aβ plaque, neurofibrillary tangles, synaptic dysfunction, neurotransmitter imbalance, neuroinflammation, gut microbiome disruption, oxidative stress, and autophagy. This range of causes reflects the heterogeneity of AD patients [93], which increases the difficulty of exploring identical biomarkers for different AD cases. Exosomes released by cells into circulation, as well as bodily fluids, show various protein and RNA contents in healthy subjects and patients, which can be measured as potential diagnostic markers [94]. In this review, we introduced the alteration of plasma exosomal APP and Tau cleavage products as a potential biomarker for different AD stages; however, more specific and precise biomarkers of the different causes of AD cases still require further exploration.

This review also described how the exosomes extracted from bodily fluids, including CSF, plasma, urinary, or salivary exosomes, carry various diagnostic molecules, such as Aβ_40_, Aβ_42_, tTau, and p-Tau, which are also considered as biomarkers of CSF in AD diagnosis. Exosomes may provide more exact data to identify early markers of AD based on their precisely regulated biogenetic processes and structural features. However, a lack of standard and efficient isolation and characterization techniques places significant constraints on the use of exosomes as a tool in AD diagnosis. Currently, differential ultracentrifugation is considered the gold standard for separating exosomes; however, this method is time-consuming. Other methods, such as immunoprecipitation and size filtration, have been developed to avoid the need for ultracentrifugation. However, these methods typically lead to high levels of polluted proteins and a mixture of extracellular vesicles. Thus, extraction technology, identification standardization, and database construction of exosomes need to be the further developed, as this will provide a new basis for the early detection of exosomal APP cleavage products or p-Tau in AD.

## Figures and Tables

**Table 1:** Proteins that participate in regulation of the release of exosomes containing APP or Tau cleavage products.

Protein	Promotes or Inhibits Exosome Release	Major Contents of Exosome	References
PI3K-dependent pathway	Promotes	APP and its cleaved products	[40]
nSMase2	Inhibits	Aβ_42_	[41,42]
PrP^C^	Balances	Aβ_42_	[43,44]
GAC	Promotes	APP and Aβ	[45]
CaSR antagonist NPS2143	Inhibits	Tau and p-Tau	[46]

PI3K: phosphoinositide 3-kinase; nSMase2: neutral sphingomyelinase 2; PrP^C^: cellular prion protein; GAC: glutaminase C; CaSR: calcium-sensing receptor.

## Data Availability

Not applicable.

## References

[1] Jia J., Wei C., Chen S., Li F., Tang Y., Qin W., Zhao L., Jin H., Xu H., Wang F. (2018). The cost of Alzheimer’s disease in China and re-estimation of costs worldwide. Alzheimers Dement..

[2] Haukedal H., Freude K.K. (2020). Implications of Glycosylation in Alzheimer’s Disease. Front. Neurosci..

[3] Wang J., Gu B.J., Masters C.L., Wang Y.J. (2017). A systemic view of Alzheimer disease—Insights from amyloid-beta metabolism beyond the brain. Nat. Rev. Neurol..

[4] Lakshmi S., Essa M.M., Hartman R.E., Guillemin G.J., Sivan S., Elumalai P. (2020). Exosomes in Alzheimer’s Disease: Potential Role as Pathological Mediators, Biomarkers and Therapeutic Targets. Neurochem. Res..

[5] Mohamed T., Shakeri A., Rao P.P. (2016). Amyloid cascade in Alzheimer’s disease: Recent advances in medicinal chemistry. Eur. J. Med. Chem..

[6] Suh Y.H., Checler F. (2002). Amyloid precursor protein, presenilins, and alpha-synuclein: Molecular pathogenesis and pharmacological applications in Alzheimer’s disease. Pharmacol. Rev..

[7] Lei Y., Renyuan Z. (2018). Effects of Androgens on the Amyloid-β Protein in Alzheimer’s Disease. Endocrinology.

[8] Tiwari S., Atluri V., Kaushik A., Yndart A., Nair M. (2019). Alzheimer’s disease: Pathogenesis, diagnostics, and therapeutics. Int. J. Nanomed..

[9] Behl T., Kaur I., Fratila O., Brata R., Bungau S. (2020). Exploring the Potential of Therapeutic Agents Targeted towards Mitigating the Events Associated with Amyloid-β Cascade in Alzheimer’s Disease. Int. J. Mol. Sci..

[10] Barage S.H., Sonawane K.D. (2015). Amyloid cascade hypothesis: Pathogenesis and therapeutic strategies in Alzheimer’s disease. Neuropeptides.

[11] Shankar G.M., Li S., Mehta T.H., Garcia-Munoz A., Shepardson N.E., Smith I., Brett F.M., Farrell M.A., Rowan M.J., Lemere C.A. (2008). Amyloid-beta protein dimers isolated directly from Alzheimer’s brains impair synaptic plasticity and memory. Nat. Med..

[12] Lee H.E., Lim D., Lee J.Y., Lim S.M., Pae A.N. (2019). Development of tau-directed small molecule modulators for Alzheimer’s disease: A recent patent review (2014-2018). Pharm. Pat. Anal..

[13] Naseri N.N., Wang H., Guo J., Sharma M., Luo W. (2019). The complexity of tau in Alzheimer’s disease. Neurosci. Lett..

[14] Hanger D.P., Seereeram A., Noble W. (2009). Mediators of tau phosphorylation in the pathogenesis of Alzheimer’s disease. Expert Rev. Neurother..

[15] Hanger D.P., Anderton B.H., Noble W. (2009). Tau phosphorylation: The therapeutic challenge for neurodegenerative disease. Trends Mol. Med..

[16] Gratuze M., Joly-Amado A., Vieau D., Buée L., Blum D. (2018). Mutual Relationship between Tau and Central Insulin Signalling: Consequences for AD and Tauopathies?. Neuroendocrinology.

[17] Maccioni R.B., Farías G., Morales I., Navarrete L. (2010). The revitalized tau hypothesis on Alzheimer’s disease. Arch. Med. Res..

[18] Gao Y., Tan L., Yu J.T., Tan L. (2018). Tau in Alzheimer’s Disease: Mechanisms and Therapeutic Strategies. Curr. Alzheimer Res..

[19] Cooper L.F., Ravindran S., Huang C.C., Kang M. (2019). A Role for Exosomes in Craniofacial Tissue Engineering and Regeneration. Front. Physiol..

[20] Kalluri R., LeBleu V.S. (2020). The biology, function, and biomedical applications of exosomes. Science.

[21] Mashouri L., Yousefi H., Aref A.R., Ahadi A.M., Molaei F., Alahari S.K. (2019). Exosomes: Composition, biogenesis, and mechanisms in cancer metastasis and drug resistance. Mol. Cancer.

[22] Feng J., Zhang Y., Zhu Z., Gu C., Waqas A., Chen L. (2021). Emerging Exosomes and Exosomal MiRNAs in Spinal Cord Injury. Front. Cell Dev. Biol..

[23] Yuan Z., Huang W. (2021). New Developments in Exosomal lncRNAs in Cardiovascular Diseases. Front. Cardiovasc. Med..

[24] Heidarzadeh M., Gürsoy-Özdemir Y., Kaya M., Eslami Abriz A., Zarebkohan A., Rahbarghazi R., Sokullu E. (2021). Exosomal delivery of therapeutic modulators through the blood-brain barrier; promise and pitfalls. Cell Biosci..

[25] van Niel G., D’Angelo G., Raposo G. (2018). Shedding light on the cell biology of extracellular vesicles. Nat. Rev. Mol. Cell Biol..

[26] Riau A.K., Ong H.S., Yam G.H.F., Mehta J.S. (2019). Sustained Delivery System for Stem Cell-Derived Exosomes. Front. Pharmacol..

[27] Eitan E., Suire C., Zhang S., Mattson M.P. (2016). Impact of lysosome status on extracellular vesicle content and release. Ageing Res. Rev..

[28] Lötvall J., Hill A.F., Hochberg F., Buzás E.I., Di Vizio D., Gardiner C., Gho Y.S., Kurochkin I.V., Mathivanan S., Quesenberry P. (2014). Minimal experimental requirements for definition of extracellular vesicles and their functions: A position statement from the International Society for Extracellular Vesicles. J. Extracell. Vesicles.

[29] Poliakov A., Spilman M., Dokland T., Amling C.L., Mobley J.A. (2009). Structural heterogeneity and protein composition of exosome-like vesicles (prostasomes) in human semen. Prostate.

[30] van der Pol E., Coumans F.A., Grootemaat A.E., Gardiner C., Sargent I.L., Harrison P., Sturk A., van Leeuwen T.G., Nieuwland R. (2014). Particle size distribution of exosomes and microvesicles determined by transmission electron microscopy, flow cytometry, nanoparticle tracking analysis, and resistive pulse sensing. J. Thromb. Haemost. JTH.

[31] Thej C., Kishore R. (2021). Unfathomed Nanomessages to the Heart: Translational Implications of Stem Cell-Derived, Progenitor Cell Exosomes in Cardiac Repair and Regeneration. Cells.

[32] Peng L., Wang D., Han Y., Huang T., He X., Wang J., Ou C. (2021). Emerging Role of Cancer-Associated Fibroblasts-Derived Exosomes in Tumorigenesis. Front. Immunol..

[33] Polanco J.C., Li C., Durisic N., Sullivan R., Götz J. (2018). Exosomes taken up by neurons hijack the endosomal pathway to spread to interconnected neurons. Acta Neuropathol. Commun..

[34] Pérez-González R., Kim Y., Miller C., Pacheco-Quinto J., Eckman E.A., Levy E. (2020). Extracellular vesicles: Where the amyloid precursor protein carboxyl-terminal fragments accumulate and amyloid-β oligomerizes. FASEB J. Off. Publ. Fed. Am. Soc. Exp. Biol..

[35] Choi Y., Kim S.M., Heo Y., Lee G., Kang J.Y., Yoon D.S. (2021). Nanoelectrical characterization of individual exosomes secreted by Aβ(42)-ingested cells using electrostatic force microscopy. Nanotechnology.

[36] Zheng T., Pu J., Chen Y., Mao Y., Guo Z., Pan H., Zhang L., Zhang H., Sun B., Zhang B. (2017). Plasma Exosomes Spread and Cluster Around β-Amyloid Plaques in an Animal Model of Alzheimer’s Disease. Front. Aging Neurosci..

[37] Zheng T., Wu X., Wei X., Wang M., Zhang B. (2018). The release and transmission of amyloid precursor protein via exosomes. Neurochem. Int..

[38] Sardar Sinha M., Ansell-Schultz A., Civitelli L., Hildesjö C., Larsson M., Lannfelt L., Ingelsson M., Hallbeck M. (2018). Alzheimer’s disease pathology propagation by exosomes containing toxic amyloid-beta oligomers. Acta Neuropathol..

[39] Laulagnier K., Javalet C., Hemming F.J., Chivet M., Lachenal G., Blot B., Chatellard C., Sadoul R. (2018). Amyloid precursor protein products concentrate in a subset of exosomes specifically endocytosed by neurons. Cell Mol. Life Sci..

[40] Wu Q., Cortez L., Kamali-Jamil R., Sim V., Wille H., Kar S. (2021). Implications of exosomes derived from cholesterol-accumulated astrocytes in Alzheimer’s disease pathology. Dis. Model Mech..

[41] Dinkins M.B., Enasko J., Hernandez C., Wang G., Kong J., Helwa I., Liu Y., Terry A.V., Bieberich E. (2016). Neutral Sphingomyelinase-2 Deficiency Ameliorates Alzheimer’s Disease Pathology and Improves Cognition in the 5XFAD Mouse. J. Neurosci. Off. J. Soc. Neurosci..

[42] Dinkins M.B., Dasgupta S., Wang G., Zhu G., Bieberich E. (2014). Exosome reduction in vivo is associated with lower amyloid plaque load in the 5XFAD mouse model of Alzheimer’s disease. Neurobiol. Aging.

[43] Falker C., Hartmann A., Guett I., Dohler F., Altmeppen H., Betzel C., Schubert R., Thurm D., Wegwitz F., Joshi P. (2016). Exosomal cellular prion protein drives fibrillization of amyloid beta and counteracts amyloid beta-mediated neurotoxicity. J. Neurochem..

[44] Qin K., Zhao L., Gregory C., Solanki A., Mastrianni J.A. (2019). “Dual Disease” TgAD/GSS mice exhibit enhanced Alzheimer’s disease pathology and reveal PrP(C)-dependent secretion of Aβ. Sci. Rep..

[45] Gao G., Zhao S., Xia X., Li C., Li C., Ji C., Sheng S., Tang Y., Zhu J., Wang Y. (2019). Glutaminase C Regulates Microglial Activation and Pro-inflammatory Exosome Release: Relevance to the Pathogenesis of Alzheimer’s Disease. Front. Cell. Neurosci..

[46] Chiarini A., Armato U., Gardenal E., Gui L., Dal Prà I. (2017). Amyloid β-Exposed Human Astrocytes Overproduce Phospho-Tau and Overrelease It within Exosomes, Effects Suppressed by Calcilytic NPS 2143-Further Implications for Alzheimer’s Therapy. Front. Neurosci..

[47] Abdullah M., Takase H., Nunome M., Enomoto H., Ito J., Gong J.S., Michikawa M. (2016). Amyloid-β Reduces Exosome Release from Astrocytes by Enhancing JNK Phosphorylation. J. Alzheimers Dis..

[48] Nafar F., Williams J.B., Mearow K.M. (2016). Astrocytes release HspB1 in response to amyloid-β exposure in vitro. J. Alzheimers Dis..

[49] Elsherbini A., Kirov A.S., Dinkins M.B., Wang G., Qin H., Zhu Z., Tripathi P., Crivelli S.M., Bieberich E. (2020). Association of Aβ with ceramide-enriched astrosomes mediates Aβ neurotoxicity. Acta Neuropathol. Commun..

[50] Deng Z., Wang J., Xiao Y., Li F., Niu L., Liu X., Meng L., Zheng H. (2021). Ultrasound-mediated augmented exosome release from astrocytes alleviates amyloid-β-induced neurotoxicity. Theranostics.

[51] Pitt J., Wilcox K.C., Tortelli V., Diniz L.P., Oliveira M.S., Dobbins C., Yu X.W., Nandamuri S., Gomes F.C.A., DiNunno N. (2017). Neuroprotective astrocyte-derived insulin/insulin-like growth factor 1 stimulates endocytic processing and extracellular release of neuron-bound Aβ oligomers. Mol. Biol. Cell.

[52] Fernandes A., Ribeiro A.R., Monteiro M., Garcia G., Vaz A.R., Brites D. (2018). Secretome from SH-SY5Y APP(Swe) cells trigger time-dependent CHME3 microglia activation phenotypes, ultimately leading to miR-21 exosome shuttling. Biochimie.

[53] Huang S., Liao X., Wu J., Zhang X., Li Y., Xiang D., Luo S. (2022). The Microglial membrane receptor TREM2 mediates exosome secretion to promote phagocytosis of amyloid-β by microglia. FEBS Lett..

[54] Han S., Kollmer M., Markx D., Claus S., Walther P., Fändrich M. (2017). Amyloid plaque structure and cell surface interactions of β-amyloid fibrils revealed by electron tomography. Sci. Rep..

[55] Williamson R.L., Laulagnier K., Miranda A.M., Fernandez M.A., Wolfe M.S., Sadoul R., Di Paolo G. (2017). Disruption of amyloid precursor protein ubiquitination selectively increases amyloid β (Aβ) 40 levels via presenilin 2-mediated cleavage. J. Biol. Chem..

[56] Lauritzen I., Pardossi-Piquard R., Bourgeois A., Pagnotta S., Biferi M.G., Barkats M., Lacor P., Klein W., Bauer C., Checler F. (2016). Intraneuronal aggregation of the β-CTF fragment of APP (C99) induces Aβ-independent lysosomal-autophagic pathology. Acta Neuropathol..

[57] Lauritzen I., Bécot A., Bourgeois A., Pardossi-Piquard R., Biferi M.G., Barkats M., Checler F. (2019). Targeting γ-secretase triggers the selective enrichment of oligomeric APP-CTFs in brain extracellular vesicles from Alzheimer cell and mouse models. Transl. Neurodegener..

[58] Cone A.S., Hurwitz S.N., Lee G.S., Yuan X., Zhou Y., Li Y., Meckes D.G. (2020). Alix and Syntenin-1 direct amyloid precursor protein trafficking into extracellular vesicles. BMC Mol. Cell Biol..

[59] Miranda A.M., Lasiecka Z.M., Xu Y., Neufeld J., Shahriar S., Simoes S., Chan R.B., Oliveira T.G., Small S.A., Di Paolo G. (2018). Neuronal lysosomal dysfunction releases exosomes harboring APP C-terminal fragments and unique lipid signatures. Nat. Commun..

[60] Xie J.C., Ma X.Y., Liu X.H., Yu J., Zhao Y.C., Tan Y., Liu X.Y., Zhao Y.X. (2018). Hypoxia increases amyloid-β level in exosomes by enhancing the interaction between CD147 and Hook1. Am. J. Transl. Res..

[61] Pacheco-Quinto J., Clausen D., Pérez-González R., Peng H., Meszaros A., Eckman C.B., Levy E., Eckman E.A. (2019). Intracellular metalloprotease activity controls intraneuronal Aβ aggregation and limits secretion of Aβ via exosomes. FASEB J. Off. Publ. Fed. Am. Soc. Exp. Biol..

[62] Guix F.X., Sannerud R., Berditchevski F., Arranz A.M., Horré K., Snellinx A., Thathiah A., Saido T., Saito T., Rajesh S. (2017). Tetraspanin 6: A pivotal protein of the multiple vesicular body determining exosome release and lysosomal degradation of amyloid precursor protein fragments. Mol. Neurodegener..

[63] Winston C.N., Goetzl E.J., Akers J.C., Carter B.S., Rockenstein E.M., Galasko D., Masliah E., Rissman R.A. (2016). Prediction of conversion from mild cognitive impairment to dementia with neuronally derived blood exosome protein profile. Alzheimer’s Dement..

[64] Polanco J.C., Scicluna B.J., Hill A.F., Götz J. (2016). Extracellular Vesicles Isolated from the Brains of rTg4510 Mice Seed Tau Protein Aggregation in a Threshold-dependent Manner. J. Biol. Chem..

[65] Baker S., Polanco J.C., Götz J. (2016). Extracellular Vesicles Containing P301L Mutant Tau Accelerate Pathological Tau Phosphorylation and Oligomer Formation but Do Not Seed Mature Neurofibrillary Tangles in ALZ17 Mice. J. Alzheimers Dis..

[66] Wang Y., Balaji V., Kaniyappan S., Krüger L., Irsen S., Tepper K., Chandupatla R., Maetzler W., Schneider A., Mandelkow E. (2017). The release and trans-synaptic transmission of Tau via exosomes. Mol. Neurodegener..

[67] Guix F.X., Corbett G.T., Cha D.J., Mustapic M., Liu W., Mengel D., Chen Z., Aikawa E., Young-Pearse T., Kapogiannis D. (2018). Detection of Aggregation-Competent Tau in Neuron-Derived Extracellular Vesicles. Int. J. Mol. Sci..

[68] Winston C.N., Aulston B., Rockenstein E.M., Adame A., Prikhodko O., Dave K.N., Mishra P., Rissman R.A., Yuan S.H. (2019). Neuronal Exosome-Derived Human Tau is Toxic to Recipient Mouse Neurons in vivo. J. Alzheimers Dis..

[69] Podvin S., Jones A., Liu Q., Aulston B., Ransom L., Ames J., Shen G., Lietz C.B., Jiang Z., O’Donoghue A.J. (2020). Dysregulation of Exosome Cargo by Mutant Tau Expressed in Human-induced Pluripotent Stem Cell (iPSC) Neurons Revealed by Proteomics Analyses. Mol. Cell. Proteom. MCP.

[70] Asai H., Ikezu S., Tsunoda S., Medalla M., Luebke J., Haydar T., Wolozin B., Butovsky O., Kügler S., Ikezu T. (2015). Depletion of microglia and inhibition of exosome synthesis halt tau propagation. Nat. Neurosci..

[71] Polanco J.C., Hand G.R., Briner A., Li C., Götz J. (2021). Exosomes induce endolysosomal permeabilization as a gateway by which exosomal tau seeds escape into the cytosol. Acta Neuropathol..

[72] Marcus C., Mena E., Subramaniam R.M. (2014). Brain PET in the diagnosis of Alzheimer’s disease. Clin. Nucl. Med..

[73] Welge V., Fiege O., Lewczuk P., Mollenhauer B., Esselmann H., Klafki H.W., Wolf S., Trenkwalder C., Otto M., Kornhuber J. (2009). Combined CSF tau, p-tau181 and amyloid-beta 38/40/42 for diagnosing Alzheimer’s disease. J. Neural Transm..

[74] Eitan E., Hutchison E.R., Marosi K., Comotto J., Mustapic M., Nigam S.M., Suire C., Maharana C., Jicha G.A., Liu D. (2016). Extracellular Vesicle-Associated Aβ Mediates Trans-Neuronal Bioenergetic and Ca(2+)-Handling Deficits in Alzheimer’s Disease Models. NPJ Aging Mech. Dis..

[75] Gibbons G.S., Lee V.M.Y., Trojanowski J.Q. (2019). Mechanisms of Cell-to-Cell Transmission of Pathological Tau: A Review. JAMA Neurol..

[76] Perrotte M., Haddad M., Le Page A., Frost E.H., Fulöp T., Ramassamy C. (2020). Profile of pathogenic proteins in total circulating extracellular vesicles in mild cognitive impairment and during the progression of Alzheimer’s disease. Neurobiol. Aging.

[77] Sun R., Wang H., Shi Y., Sun Z., Jiang H., Zhang J. (2020). Changes in the Morphology, Number, and Pathological Protein Levels of Plasma Exosomes May Help Diagnose Alzheimer’s Disease. J. Alzheimers Dis..

[78] Shi M., Kovac A., Korff A., Cook T.J., Ginghina C., Bullock K.M., Yang L., Stewart T., Zheng D., Aro P. (2016). CNS tau efflux via exosomes is likely increased in Parkinson’s disease but not in Alzheimer’s disease. Alzheimers Dement..

[79] Li Y., Meng S., Di W., Xia M., Dong L., Zhao Y., Ling S., He J., Xue X., Chen X. (2022). Amyloid-β protein and MicroRNA-384 in NCAM-Labeled exosomes from peripheral blood are potential diagnostic markers for Alzheimer’s disease. CNS Neurosci. Ther..

[80] Eren E., Hunt J.F.V., Shardell M., Chawla S., Tran J., Gu J., Vogt N.M., Johnson S.C., Bendlin B.B., Kapogiannis D. (2020). Extracellular vesicle biomarkers of Alzheimer’s disease associated with sub-clinical cognitive decline in late middle age. Alzheimers Dement..

[81] Abner E.L., Jicha G.A., Shaw L.M., Trojanowski J.Q., Goetzl E.J. (2016). Plasma neuronal exosomal levels of Alzheimer’s disease biomarkers in normal aging. Ann. Clin. Transl. Neurol..

[82] Jia L., Qiu Q., Zhang H., Chu L., Du Y., Zhang J., Zhou C., Liang F., Shi S., Wang S. (2019). Concordance between the assessment of Aβ_42_, T-tau, and P-T181-tau in peripheral blood neuronal-derived exosomes and cerebrospinal fluid. Alzheimers Dement..

[83] Zhao A., Li Y., Yan Y., Qiu Y., Li B., Xu W., Wang Y., Liu J., Deng Y. (2020). Increased prediction value of biomarker combinations for the conversion of mild cognitive impairment to Alzheimer’s dementia. Transl. Neurodegener..

[84] Porsteinsson A.P., Isaacson R.S., Knox S., Sabbagh M.N., Rubino I. (2021). Diagnosis of Early Alzheimer’s Disease: Clinical Practice in 2021. J. Prev. Alzheimer’s Dis..

[85] Soares Martins T., Marçalo R., da Cruz E.S.C.B., Trindade D., Catita J., Amado F., Melo T., Rosa I.M., Vogelgsang J., Wiltfang J. (2022). Novel Exosome Biomarker Candidates for Alzheimer’s Disease Unravelled Through Mass Spectrometry Analysis. Mol. Neurobiol..

[86] Soares Martins T., Marçalo R., Ferreira M., Vaz M., Silva R.M., Martins Rosa I., Vogelgsang J., Wiltfang J., da Cruz E.S.O.A.B., Henriques A.G. (2021). Exosomal Aβ-Binding Proteins Identified by “In Silico” Analysis Represent Putative Blood-Derived Biomarker Candidates for Alzheimer’s Disease. Int. J. Mol. Sci..

[87] Wang D., Wang P., Bian X., Xu S., Zhou Q., Zhang Y., Ding M., Han M., Huang L., Bi J. (2020). Elevated plasma levels of exosomal BACE1-AS combined with the volume and thickness of the right entorhinal cortex may serve as a biomarker for the detection of Alzheimer’s disease. Mol. Med. Rep..

[88] Goetzl E.J., Mustapic M., Kapogiannis D., Eitan E., Lobach I.V., Goetzl L., Schwartz J.B., Miller B.L. (2016). Cargo proteins of plasma astrocyte-derived exosomes in Alzheimer’s disease. FASEB J. Off. Publ. Fed. Am. Soc. Exp. Biol..

[89] Cohn W., Melnik M., Huang C., Teter B., Chandra S., Zhu C., McIntire L.B., John V., Gylys K.H., Bilousova T. (2021). Multi-Omics Analysis of Microglial Extracellular Vesicles From Human Alzheimer’s Disease Brain Tissue Reveals Disease-Associated Signatures. Front. Pharmacol..

[90] Delgado-Peraza F., Nogueras-Ortiz C.J., Volpert O., Liu D., Goetzl E.J., Mattson M.P., Greig N.H., Eitan E., Kapogiannis D. (2021). Neuronal and Astrocytic Extracellular Vesicle Biomarkers in Blood Reflect Brain Pathology in Mouse Models of Alzheimer’s Disease. Cells.

[91] Sun R., Wang H., Shi Y., Gao D., Sun Z., Chen Z., Jiang H., Zhang J. (2019). A Pilot Study of Urinary Exosomes in Alzheimer’s Disease. Neuro-Degener. Dis..

[92] Rani K., Rastogi S., Vishwakarma P., Bharti P.S., Sharma V., Renu K., Modi G.P., Vishnu V.Y., Chatterjee P., Dey A.B. (2021). A novel approach to correlate the salivary exosomes and their protein cargo in the progression of cognitive impairment into Alzheimer’s disease. J. Neurosci. Methods.

[93] Khan S., Barve K.H., Kumar M.S. (2020). Recent Advancements in Pathogenesis, Diagnostics and Treatment of Alzheimer’s Disease. Curr. Neuropharmacol..

[94] Barile L., Vassalli G. (2017). Exosomes: Therapy delivery tools and biomarkers of diseases. Pharmacol. Ther..

